# Efficient On‐Column Removal of Endotoxin from Immunoglobulins Such as AK23

**DOI:** 10.1002/cpz1.70238

**Published:** 2025-11-06

**Authors:** Siavash Rahimi, Patrizia Sauta, Monika Edler, Elisabeth Locher, Marlies Illi, Taravat Shojaeian, Luca Borradori, Thomas Gentinetta, William V. J. Hariton, Eliane J. Müller

**Affiliations:** ^1^ Department for Biomedical Research, Molecular Dermatology and Stem Cell Research University of Bern Bern Switzerland; ^2^ Department of Dermatology, Inselspital, Bern University Hospital University of Bern Bern Switzerland; ^3^ Graduate School Cellular and Biomedical Sciences University of Bern Bern Switzerland; ^4^ CSL, CSL Biologics Research Centre Bern Switzerland; ^5^ Swiss Institute for Translational and Entrepreneurial Medicine, sitem‐insel Bern Switzerland

**Keywords:** autoimmune bullous disease, antibody, endotoxin, inflammation

## Abstract

Unattended endotoxin (ETX) contamination in biological samples constitute a major challenge for *in vitro* and *in vivo* applications. Besides being potentially life‐threatening, ETX contamination is especially relevant in global transcriptome analyses, where competing ETX stimulation can significantly skew the final gene expression profile. Our studies in mice and cultured skin epithelial cells (epidermal keratinocytes) aiming to characterize the effect of antibodies such as AK23 immunoglobulins (IgG directed against the cell‐cell adhesion molecule desmoglein [DSG] 3) in the autoimmune disease pemphigus vulgaris (PV) revealed that laboratory‐produced and even commercial control antibodies can exhibit non‐negligible ETX contaminations. Moreover, these contaminants are extremely difficult to remove. To overcome these challenges, we have devised a simple yet nontoxic and scalable two‐step protocol to efficiently reduce ETX levels during or after the IgG purification process. It consists firstly of 0.5 M NaOH pre‐treatment of all devices, including the protein A resin, used during IgG sanitization and purification, in parallel with meticulous in‐process monitoring of ETX levels. Secondly, before IgG elution from protein A, ETX is stripped from IgG by ion‐exchange with the common amino acid arginine. This two‐step approach successfully reduces ETX by >95% from hybridoma‐derived, laboratory‐produced AK23 IgG, as well as patient PV and control IgG, resulting in an 85% IgG recovery rate and ETX levels compatible with *U.S. Pharmacopeia* guidelines. © 2025 The Author(s). *Current Protocols* published by Wiley Periodicals LLC.

**Basic Protocol 1**: Sanitization of devices and protein A resin with 0.5 m naoh

**Basic Protocol 2**: On‐column stripping of ETX from AK23 IgG

**Basic Protocol 3**: Quality control of sanitized AK23 IgG

## INTRODUCTION

Endotoxins (ETXs) are lipopolysaccharides (LPS) released from the outer cell walls of live and dead Gram‐negative bacteria. ETX is omnipresent and heat stable, and hence survives standard sterilization conditions for devices and buffers. ETX binds to CD14 and Toll‐like receptor 4 (TLR4), which are predominantly expressed on myeloid cells (Gorbet & Sefton, 2005). Additionally, bacterial contaminants can engage TLR2. Non‐leukocytic cells, including epidermal keratinocytes, also express these immunoreactive receptors and respond to ETX (Song et al., 2002) with enhanced expression of cytokines such as TNF‐α, NF‐κB, IL‐1β, IL‐6, and IL‐8, as reported in 2D cultures and human skin explants (Gvirtz et al., 2020; Kock et al., 1990). Notably, LPS has also been shown to trigger an increase in intracellular calcium ([Ca²⁺]_i_) levels in cultured human keratinocytes through CD14 and TLR4 signaling and to enhance proliferation (Sugita et al., 1986a) and keratin expression (Sugita et al., 1986b) of basal keratinocytes. Moreover, co‐stimulation with LPS and other inflammatory mediators, such as prostaglandin E2, skews the response of keratinocytes by inverting an anti‐proliferative into a pro‐proliferative response (Sugita et al., 1986a). This underscores the importance of the impact of unattended ETX contaminations in cells and tissues of non‐myeloid origin (Heinrich et al., 2022). It further emphasizes the critical need for rigorous ETX monitoring, as described previously (Müller et al., 2024), as well as the necessity of removing ETX from biological samples, whether injected into humans and mice or used in cell culture with sensitive read‐outs, such as transcriptomics and proteomics.

We have previously established a protocol to produce the experimental monoclonal antibody AK23 from hybridoma cells (Tsunoda et al., 2003) with reproducible activity for *in vivo* (pemphigus vulgaris [PV] mouse models) and *in vitro* (cultured mouse and human keratinocytes) modeling of PV (Müller et al., 2024). PV is a potentially life‐threatening autoimmune disease of the skin and mucous membranes in which autoantibodies target either one or two adhesion molecules of the desmosomal cadherin family, that is, either desmoglein (DSG) 3 or DSG3 and DSG1 simultaneously. This leads to severe, potentially life‐threatening blistering of mucous membranes and skin, respectively (di Zenzo et al., 2016; Hammers & Stanley, 2016; Rahimi et al., 2025; Schmidt et al., 2019). The disease‐initiating signals, as well as the transcriptomic alterations that occur upon autoantibody binding to the keratinocyte surface in PV *in vivo* and *in vitro* models (Hartmann et al., 2023), are the focus of intense research aimed at identifying novel first‐line treatments for PV (Müller et al., 2008; Rahimi et al., 2025; Schmitt & Waschke, 2021). Similarly to what has been demonstrated for ETX, described above (Gvirtz et al., 2020; Kock et al., 1990), PV antibody binding to the skin keratinocyte surface can upregulate the expression of inflammatory cytokines such as IL‐17 and TNF‐α but also activate NF‐κB (Assaf et al., 2021; Feliciani et al., 2000; Godsel et al., 2022; Holstein et al., 2021; Liang et al., 2017; Orlov et al., 2006; Rahimi et al., 2025; Wang et al., 2024), and enhances proliferation of basal keratinocytes (Schulze et al., 2012; Williamson et al., 2006), which might play an important role in triggering the disease. Low‐ETX or ETX‐free conditions are thus a prerequisite to exclude confounding ETX‐triggered signals that could skew specific, antibody‐induced cellular responses.

Monitoring the ETX levels in antibody preparations in general, and in AK23 IgG, PV IgG, and control IgG in particular, is considered good laboratory practice as a prerequisite to injecting IgG into the PV mouse model (Hariton et al., 2017; Hariton et al., 2023; Luyet et al., 2015; Schulze et al., 2012). ETX monitoring is also advised when addressing sensitive parameters other than end stage (e.g., in PV, loss of intercellular adhesion) with advanced technologies such as omics analyses of cultured keratinocytes. To monitor ETX levels, the *Limulus* amebocyte lysate (LAL) assay, with a sensitivity range of 0.05 to 500 ETX units (EU)/ml, is routinely used (Ong et al., 2006; Schneier et al., 2020), and we have described its application in the context of AK23 production (Müller et al., 2024). Regulatory guidelines such as those of the *U.S. Pharmacopeia* specify a permissible ETX limit for non‐intrathecal injection into mice and humans below 0.005 EU per gram of body weight per hour (EU/g/h) (Malyala & Singh, 2008). For an adult mouse weighing 20 g, this equates to a maximum of 0.1 EU per single injection. In our established mouse passive transfer model of PV, AK23 is administered at a dose of 12.5 µg/g body weight to induce mucosal and hair follicle blisters (Hariton et al., 2017; Hariton et al., 2023; Luyet et al., 2015; Schulze et al., 2012). The ETX limit for AK23 (and control IgG) is therefore set at ≤0.4 EU/mg IgG, a limit that is, by extrapolation, also a reasonable target for PV‐antibody‐stimulated cultured keratinocytes and explant cultures (Heinrich et al., 2022).

**Figure 1:**
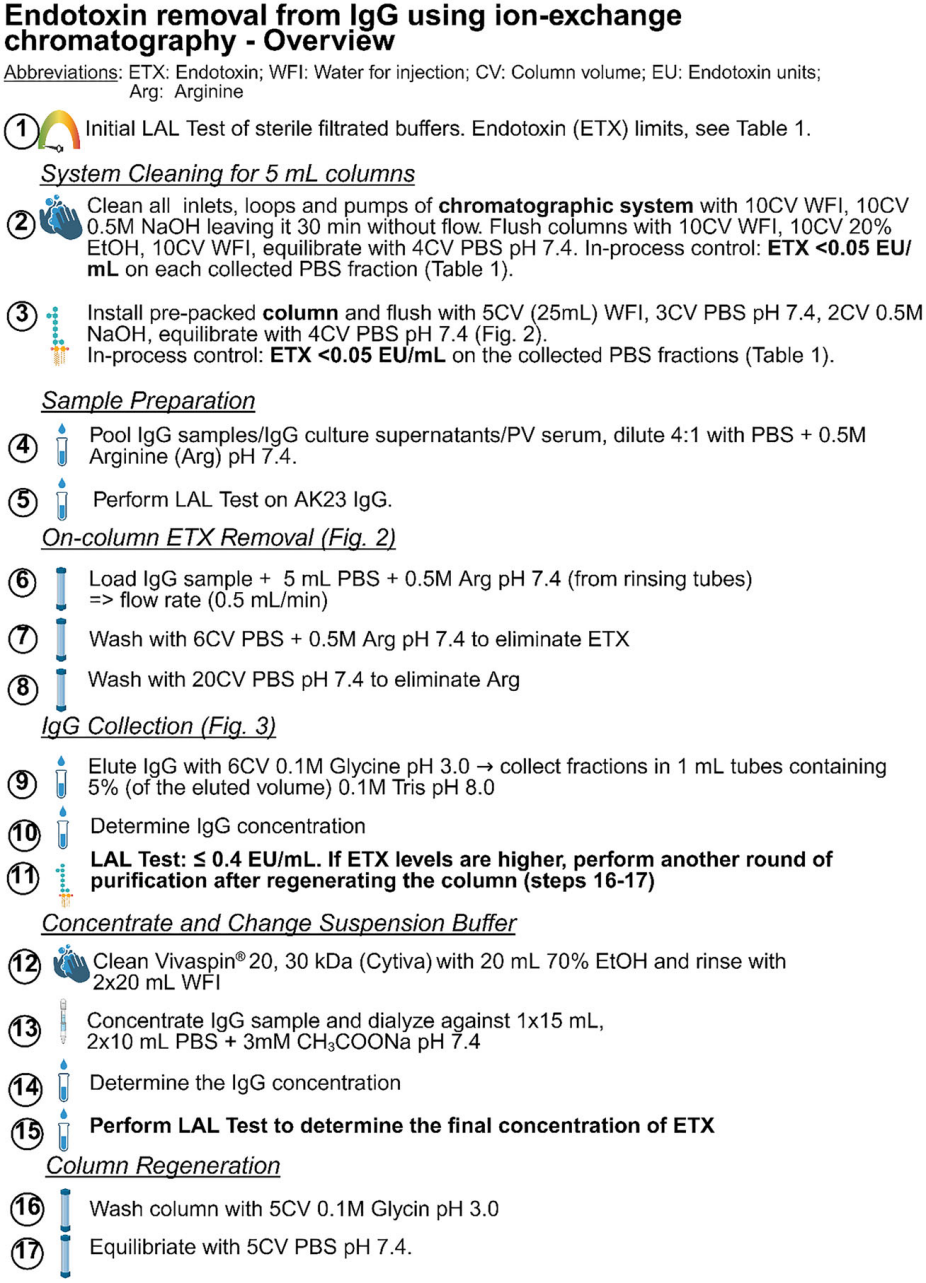
Overview of the IgG sanitization procedure. Created in BioRender (Müller, E.J., 2025; https://BioRender.com/f0fv9ee).

When ETX levels in IgG preparations exceed the necessary threshold, sanitization of the end‐product is required, but this presents a major challenge due to the high affinity of the negatively charged ETX for positively charged proteins in general and IgG in particular (Dullah & Ongkudon, 2017; Schneier et al., 2020). Although ETX removal kits, mainly based on ETX affinity chromatography, are commercially available, we found that they only reduce ETX levels in AK23 by 40%‐60% (data not shown), which is usually insufficient to comply with the stringent conditions for injection into mice (Malyala & Singh, 2008). A variety of alternative approaches with variable effectiveness and substrate specificity have been proposed, which may, however, contaminate the preparation with undesirable substances such as detergents (Aida & Pabst, 1990; Schneier et al., 2020). With the focus of devising a simple, yet nontoxic, highly effective, and scalable strategy to remove ETX from AK23 and other IgG preparations in PV research, we tested and developed a two‐step sanitization process, which is applicable to IgGs that are already purified, need to be purified from hybridoma culture supernatant (such as AK23), or are isolated from serum (such as PV IgG). This methodology is provided in the protocols below. Basic Protocol 1 outlines the first key step, which is rarely described when discussing IgG sanitization. It was guided by the pharmaceutical manufacturing process used to avoid ETX contamination (Grönberg & Hjorth, 2018) and consists of the thorough cleaning of all devices, pumps, and glassware, including the protein A resin, with 0.5 M NaOH before ETX stripping from IgG. The second step (Basic Protocol 2) is an ion‐exchange chromatography process that strips the majority of ETX from protein A‐bound IgG, which can be directly integrated into the conventional IgG isolation protocol. The stripping procedure, as exemplified here for ETX removal from AK23 IgG on an Äkta avant chromatography system, consists of ion exchange with 0.5 M arginine (a natural, nontoxic amino acid) in PBS to remove ETX. Finally, Basic Protocol 3 outlines the control of efficiency of the sanitized AK23 IgG. An overview of the full procedure is provided in Figure 1.

## Basic Protocol 1 SANITIZATION OF DEVICES AND PROTEIN A RESIN with 0.5 m naoh

Basic Protocol 1 describes in detail the mandatory preparation steps before AK23 IgG stripping from ETX from already isolated AK23 IgG or during isolation of AK23 IgG from hybridoma supernatant. We recommend using 0.5 M NaOH to sanitize (1) the glassware as well as the chromatography system used for AK23 (or other IgG) purification and (2) the protein A resin; this is followed by (3) in‐process control of ETX removal from devices and buffers.

As an alternative to the colorimetric LAL test listed below, the clotting‐based LAL test from Lonza (cat. no. N283‐06) or equivalent can be used as described in Müller et al. (2024).

*IMPORTANT NOTE*: All solutions must be prepared with water for injection (WFI) and tested for low‐level ETX contamination before use.

### Materials

Water for injection (WFI; B. Braun Medical AG, Sempach, cat. no. 45794, or equivalent)
0.5 M NaOH (Merck Supelco, cat. no. 1.09138.1000, or equivalent)
Colorimetric LAL test:
  Endosafe nexgen‐MSC benchtop (Fisher Scientific Charles River, cat. no. 19121107/1)
  Endosafe Compendial LAL Cartridges, 0.1, 10/pk (Fisher Scientific Charles River, cat. no. NC9667085
20% (v/v) ethanol (prepared from ethanol absolute for analysis, Merck Supelco, cat. no. 1.00983, or equivalent)
Phosphate‐buffered saline (PBS), pH 7.4, sterilized using a 0.2‐µm‐pore‐size filter (Müller et al., 2024; Bichsel Switzerland, cat. no. 1100176, or equivalent)

**Figure 2:**
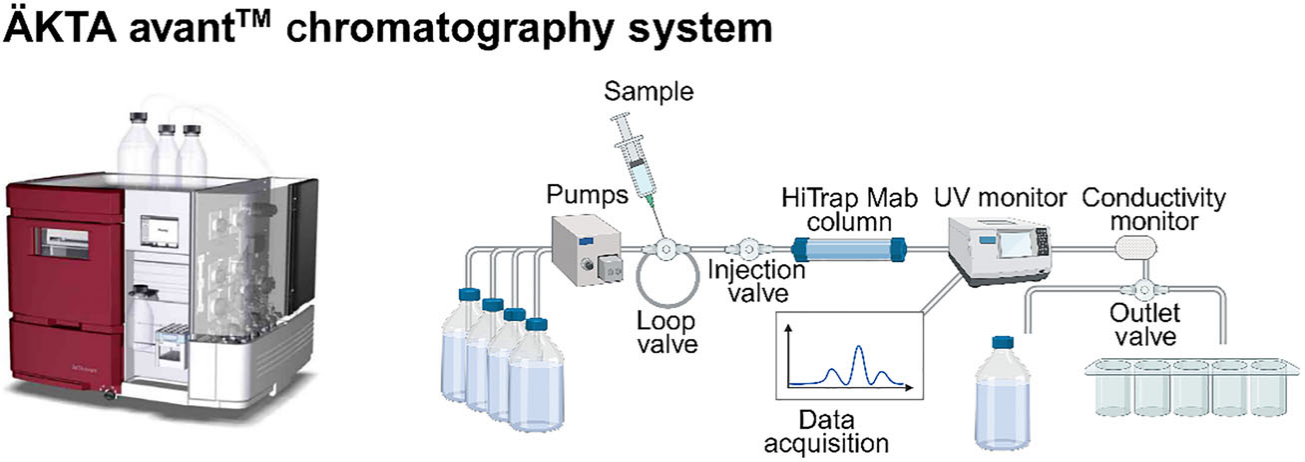
Schematic of ÄKTA avant chromatography set‐up. Created in BioRender (Müller, E., 2025; https://BioRender.com/h4aebbh).

Chromatography system: ÄKTA Avant Chromatography System (Cytiva; Fig. 2), or any other protein A column system
MabSelect Protein A HiTrap prepacked column (containing 5 ml recombinant protein A agarose; Cytiva Merck cat. no. GE17‐0403‐01) or any other NaOH‐resistant protein A matrix column

1 Prepare all solutions to be used for ETX removal and IgG purification (i.e., for Basic Protocols 1 and 2; see Table 1) with WFI, and test them using the chromogenic LAL test (or similar; see Müller et al., 2024) to ensure that ETX levels do not exceed generally accepted in‐process limits.
  Glassware used to prepare solutions must either be treated with 0.5 M NaOH and rinsed thoroughly before use or be dry heated at 250°C for 2 hr—see step 2.

**Table 1:** In‐process ETX Limits (EU/ml) for Buffers

Product	In‐process limit (EU/ml)	Result (EU/ml)
Water for injection (WFI)	<0.06	<0.01
PBS, pH 7.4	<0.2	<0.2
0.5 M arginine in PBS, pH 7.4	<0.5	<0.5
0.1 M glycine, pH 3.0	<0.5	<0.5
0.1 M Tris•Cl, pH 8.0	<0.2	<0.2
3 mM sodium acetate (NaOAc) in PBS, pH 7.4	<0.2	<0.06

**Figure 3:**
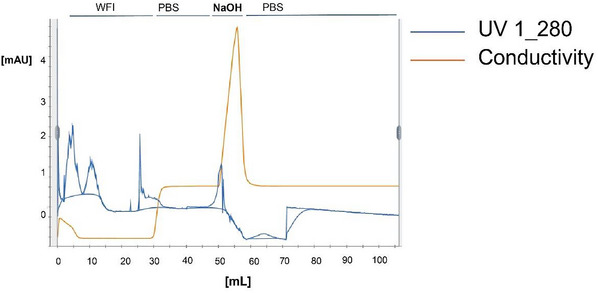
Flowthrough analysis during the sanitization of the MabSelect Protein A HiTrap column before IgG loading. The UV monitor measures protein concentrations based on *A*_260_/*A*_280_, and the conductivity monitor measures ion concentrations (Fig. 2).

2 Clean all instrument parts (including pumps and hoses) of the chromatography system (Figs. 2 and 3) with 10 column volumes of WFI and then 10 column volumes of 0.5 M NaOH, leaving the latter to react for 30 min without flow. Thoroughly flush with 10 column volumes of WFI, 10 column volumes of 20% ethanol, and 10 column volumes of WFI, and then equilibrate with 4 column volumes of PBS, pH 7.4.
  Please note that we present results obtained using a highly professional elution system suitable for in process surveillance. However, we have also successfully used the current protocol with routine laboratory equipment, such as the disposable polypropylene columns described in Müller et al. (2024), to obtain comparable results. Any chromatography column/system can be used to implement the protocol described herein. If disposable plastic columns are used, the NaOH treatment of the columns can be omitted; however, it is mandatory to clean re‐used inlet and outlet hoses as well as pumps, which, in our experience, are major sources of ETX contamination. All prescribed volumes for washing, cleaning, and neutralizing the system must, however, be strictly followed.

3 Install the pre‐packed 5‐ml MabSelect Protein A HiTrap column on the chromatography system. Flush with 5 column volumes of WFI, 3 column volumes of PBS, pH 7.4, and 2 column volumes of 0.5 M NaOH, leaving the latter to react for 30 min without flow. Equilibrate the column with 4 column volumes of PBS, pH 7.4 (Figs. 1 and 2), Collect all PBS fractions.
  Ensure that the chosen protein A resin is compatible with 0.5 M NaOH treatment.
4 Perform in‐process ETX control using the chromogenic LAL test (or similar). Ensure that the ETX level is <0.5 EU/ml in all fractions before proceeding (Table 1). If the ETX levels are too high, step 3 must be repeated.

## Basic Protocol 2 ON‐COLUMN STRIPPING OF ETX FROM AK23 IgG

Basic Protocol 2 describes ion‐exchange chromatography (with a sanitized system and low‐level ETX buffers, Basic Protocol 1 and Table 1) to remove ETX from protein A‐bound (AK23) IgG using 0.5 M arginine, which is a nontoxic, naturally occurring amino acid. Arginine has the ability to unfold protein‐protein complexes and was demonstrated previously, among many other approaches (Schneier et al., 2020), to be particularly suitable for removing ETX from IgG preparations (Ritzen et al., 2007). This step is performed on protein A‐bound IgG before conventional elution during the IgG purification process (Müller et al., 2024).

### Materials

Purified AK23 IgG (Müller et al., 2004) other IgG such as PV IgG (in buffer or serum) or control antibodies, and hybridoma culture medium supernatants (Müller et al., 2024), all ETX low
Commercial endotoxin (ETX; Lonza Bioscience, USP Reference standard endotoxin, cat. no. E700, or equivalent)
3 mM sodium acetate (CH_3_COONa; Merck, cat. no. S‐5636) in PBS, pH 7.4, <0.2 EU/ml ETX (Table 1, Basic Protocol 1)
0.5 M arginine (Fisher Scientific, cat. no. A14730.36, or equivalent) in PBS, pH 7.4, sterilized using a 0.2‐µm‐pore‐size filter, <0.5 EU/ml ETX (Table 1, Basic Protocol 1)
PBS, pH 7.4, <0.2 EU/ml ETX (Table 1, Basic Protocol 1)
0.1 M glycine, pH 3.0 (Merck, cat. no. 50046‐250g, or equivalent), <0.5 EU/ml ETX (Table 1, Basic Protocol 1)
0.1 M Tris•Cl, pH 8.0 (Merck, cat. no. T1503‐100g, or equivalent), <0.2 EU/ml ETX (Table 1, Basic Protocol 1)
70% (v/v) ethanol, diluted using water for injection (WFI) from ethanol absolute for analysis (Merck Supelco cat. no. 1.009831000), or equivalent

Chromatography system: ÄKTA Avant Chromatography System (Cytiva; Fig. 2), or any other protein A column system
MabSelect Protein A HiTrap prepacked column (containing 5 ml recombinant protein A agarose; Cytiva Merck cat. no. GE17‐0403‐01) or any other NaOH‐resistant protein A matrix column, sanitized with 0.5 M NaOH
NanoDrop spectrometer (Thermofisher Scientific, cat. no. ND‐ONE‐W) or any spectrometer measuring protein concentrations via *A*_260_/*A*_280_ ratio
20‐ml ultrafiltration columns: Vivaspin20 MWCO 3000; 20, 30 kDa (Cytiva, cat. no. 28932358)

1 Pool up to 150 mg purified AK23 IgG (in 3 mM sodium acetate in PBS, pH 7.4) or hybridoma culture medium supernatants (Müller et al., 2024) and dilute 4:1 with 0.5 M arginine in PBS, pH 7.4 (<0.5 EU/ml ETX; Table 1).
2 Load the diluted sample onto the sanitized 5‐ml MabSelect Protein A HiTrap column on the Äkta avant chromatography system. Set the flow rate to 0.5 ml/min (Fig. 3).
  The concentration of IgG does not need to be adjusted before loading the column. The binding capacity of a 5‐ml MabSelect Protein A HiTrap column can be up to 150 mg IgG.
  0.5 M arginine in PBS, pH 7.4, is used to rinse all IgG‐containing tubes.
3 Wash the column with 6 column volumes 0.5 M arginine in PBS, pH 7.4, to elute the ETX.
  Dispose of the ETX‐containing flowthrough in special waste containers for incineration.
4 Wash with 20 column volumes PBS, pH 7.4, to eliminate arginine.
5 Elute IgG with 6 column volumes of 0.1 M glycine, pH 3.0.
6 Collect fractions into 1‐ml tubes, each containing 50 µl of 0.1 M Tris•Cl, pH 8.0 (i.e., an amount equivalent to 5% of the eluted volume).
7 Determine IgG protein concentration using NanoDrop spectrometer.
  In our experience, IgG recovery after the arginine treatment is ∼85%.
8 Test IgG for ETX level using the chromogenic (or similar) LAL test (see Basic Protocol 1).
  If ETX levels are >0.4 EU/mg IgG, perform another round of purification (repeat Basic Protocols 1 and 2) after regenerating the column as described in step 12.
9 Concentrate the IgG sample to ∼1‐4 mg/ml IgG using a Vivaspin 20, 30 kDa column according to the manufacturer's protocol.
  To avoid ETX contamination, clean the Vivaspin 20 membrane once with 20 ml of 70% (v/v) ethanol and then twice with 20 ml WFI.
10 In the same Vivaspin 20 column, desalt the sample by changing the suspension buffer to 3 mM CH_3_COONa in PBS, pH 7.4, using one 15‐ml elution followed by two successive 10‐ml elutions with 3 mM CH_3_COONa in PBS, pH 7.4.
  Sodium acetate prevents IgG precipitation.
11 Determine final AK23 IgG concentration and ETX levels using the NanoDrop spectrometer and chromogenic LAL test (or similar), respectively.
  Report the final ETX level and adjust control IgG, if necessary, with commercial ETX to match the AK23 ETX levels.
12 Regenerate the protein A column by eluting it with 5 column volumes of 0.1 M glycine, pH 3.0, followed by 5 column volumes of PBS, pH 7.4.

## Basic Protocol 3 QUALITY CONTROL OF SANITIZED AK23 IgG

**Figure 4:**
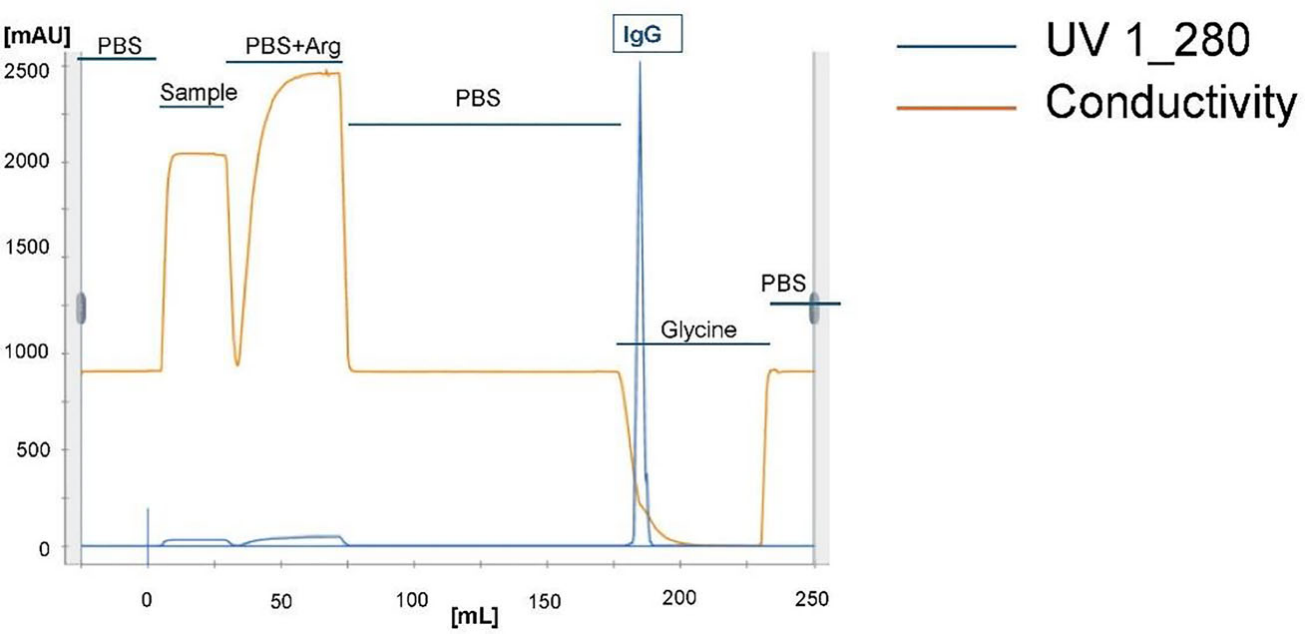
AK23 IgG purification: Flowthrough analysis for the MabSelect Protein A HiTrap column after IgG loading, depicting washing, sanitization, and elution steps. The UV monitor measures protein concentrations based on *A*_260_/*A*_280_, and the conductivity monitor measures ion concentrations (Fig. 2).

The recommended quality control procedures for sanitized AK23 consist of immunoblot analysis, SDS‐PAGE, the keratinocyte dissociation assay (KDA), and mouse passive transfer. These protocols are described in detail in the related article describing reproducible AK23 production (Müller et al., 2024). If the ETX purification procedure (Basic Protocols 1 and 2) is performed on AK23 IgG isolated from AK23 hybridoma supernatants, these quality control steps are recommended. If the ETX purification is done with already isolated AK23 IgG, controls should include validation of the functional activity of AK23 before and after sanitization using a KDA on primary human or mouse keratinocytes (Fig. 4A shows primary human keratinocytes; the method is described in detail in Müller et al., 2024). Furthermore, AK23 binding efficiency to primary human or mouse keratinocytes before and after sanitization should be tested by direct immunofluorescence (Fig. 4B shows primary human keratinocytes) as described in the protocol below.

Culture methods for primary human keratinocytes are described in Müller et al. (2024; Basic Protocol 4), except that CnT‐NX‐EX is substituted for CnT‐07 (CnT‐NX‐EX is in the same chemical family but supports long‐term expansion).

### Materials

Primary human epidermal keratinocyte progenitor (HPEK) cells (CELLnTEC, cat. no. HPEKs, lot no. ES1503029, Müller et al., 2024; human or mouse keratinocytes, including HaCat cells, are also suitable)
Materials for cell culture (see Müller et al., 2024, Basic Protocol 4)
CnT‐NX‐EX culture medium (CELLnTEC, cat. no. CnT‐NX‐EX)
226 mM CaCl_2_ stock solution (see Müller et al., 2024, Basic Protocol 4)
PBS+ (Müller et al., 2024), ice cold
1% (v/v) bovine serum albumin (BSA) in PBS+
AK23 IgG (Basic Protocol 2; IgG1 isotype; Tsunoda et al., 2003), both before and after sanitization
Isotype control mouse IgG1 (mIgG1; BioXCell, cat. no. BE0083, custom‐made, ≤0.05 EU/mg)
Methanol‐free 16% (w/v) formaldehyde (Pierce, cat. no. 28906), diluted to 4% (w/v) formaldehyde with PBS+
Goat anti‐mouse IgG (H+L) Cross‐Adsorbed Secondary Antibody, Alexa Fluor 594 (Invitrogen, cat. no. A‐11005) or equivalent
1 mg/ml Hoechst 33342 (AAT BioQuest, cat. no. 17530, or equivalent)
Fluorescence mounting medium (Dako, cat. no. S302380‐2 or equivalent)

8‐well removable chambered glass slides (ibidi, cat. no. 80841, or equivalent)
24 × 60‐mm rectangular glass cover slips, no. 1 (Corning, cat. no. CLS2975246, or equivalent)
37°C, 5% CO_2_ incubator, set to 90% humidity
Orbital shaker (Rotamax 120, P/N 544‐41200‐00, Heidolph, or equivalent)
Fluorescence microscope (Nikon, cat. no. Eclipse Ti‐E, or equivalent)

*NOTE*: It is important to perform direct immunofluorescence staining with AK23 IgG under high‐calcium conditions without fixation and at 4°C, as AK23 does not bind to fixed cells or under low‐calcium conditions, and the low temperature prevents its internalization.

1 To establish an ∼85% confluent 2D culture, seed HPEKs at 150,000 cells/cm^2^ in triplicate on chambered glass slides in 400 µl of CnT‐NX‐EX medium. Incubate overnight in a 37°C, 5% CO_2_ incubator set to 90% humidity, to allow cells to adhere and form cell‐cell contacts.
2 The following day, replace the medium with high‐calcium medium (containing 1.2 mM CaCl_2_) and incubate a further 6 hr.
  CnT‐NX‐EX contains 0.07 mM CaCl_2_, and hence an additional 1.13 mM needs to be added using the 226 mM CaCl_2_ stock solution.
3 Wash cells three times with ice‐cold PBS+.
4 Add 200 µl ice‐cold 1% BSA in PBS+ containing either the isotype control mouse IgG1 or AK23 (at an optimal dose, typically 20 µg/ml, or according to the established optimal KDA dose) to each well.
5 Incubate 1 hr at 4°C on an orbital shaker.
6 Wash cells three times with ice‐cold PBS+.
7 Add 200 µl of 4% formaldehyde and incubate 10 min at room temperature.
8 Wash cells three times, for 5 min each, in PBS+ with gentle mixing on the orbital shaker at room temperature.
9 Incubate with 200 µl goat anti‐mouse antibody, diluted 1:1000 in 1% BSA in PBS+, for 1.5 hr at room temperature with gentle mixing on an orbital shaker.
10 Add 200 µl Hoechst 33342 (1:500 in PBS+) on top of the anti‐mouse antibody solution, for a final concentration of 1:1000, and incubate for 5 min at room temperature.
  Because the cells are not permeabilized, it is important to use a lipophilic nuclear dye, such as Hoechst 33342, which can penetrate the membrane.
11 Wash cells three times, for 5 min each, in PBS+ with gentle mixing on the orbital shaker at room temperature.
12 Embed the sample in fluorescence mounting medium, cover with a coverslip, and allow the mounting medium to solidify (typically overnight at 4°C). Examine under a fluorescence microscope.
  Figure 4B shows the binding pattern of AK23 to human keratinocytes before and after sanitization. Note that you should see a comparable pattern.

## COMMENTARY

### Background Information

Endotoxin (ETX) contaminations in injectable pharmaceutical products are pyrogenic (fever‐inducing), with potential adverse outcome for humans and animals that include sepsis, organ failure, shock, and even death from as little as 1 ng per kg body weight per hour (Baird et al., 2000; de Rosa & Villa, 2023; Schneier et al., 2020). Endotoxin is composed of lipopolysaccharides and other debris present in the outer membrane of mainly Gram‐negative bacteria, which are released by living bacteria or after bacterial lysis and death. The difficulty of avoiding ETX contaminations when producing pharmacological substances is based on the fact that bacteria, and hence ETXs, are naturally present and ubiquitous. Moreover, they are highly stable and thus not destroyed or removed by conventional high‐temperature sterilization or filtration. Indeed, heat sterilization kills bacteria, likely increasing the ETX levels in the solution. It is often mistakenly believed that “sterile” solutions are also “sanitized,” and hence free of bioburden, including ETXs. This assumption can have important detrimental outcomes, as outlined above.

Over the last 15 to 20 years, cost‐effective pharmaceutical manufacturing has increasingly involved recombinant proteins produced in living organisms, in particular Gram‐negative bacteria such as *Escherichia coli* (Baird et al., 2000; Grönberg & Hjorth, 2018; Schneier et al., 2020). These biopharmaceuticals necessitated the development of (1) rapid and reliable ETX detection methods (Baker et al., 2023; Schneier et al., 2020); (2) the design of measures for efficient cleaning and sanitization of devices and buffers for purification of proteins and other pharmaceutical reactives (Grönberg & Hjorth, 2018; Schneier et al., 2020); and (3) sanitization protocols for ETX‐contaminated final products (Schneier et al., 2020). The *Limulus* amebocyte lysate (LAL) assay, which was developed in the 1960s, uses clotting factors extracted from the blood of the horseshoe crab (clotting, chromogenic, and turbidimetric methods). Although still most widely used, the LAL test is being replaced by recombinant bacterial ETX tests (Baker et al., 2023). During the production of biopharmaceuticals, regulatory bodies recommend efficient cleaning and sanitization of devices and reagents used for manufacturing, for which 0.5 to 1 M NaOH alone or in combination with other agents is the most commonly recommended (Grönberg & Hjorth, 2018). Lastly, if the end‐product exhibits ETX levels above critical limits, sanitization is required, but this represents a major challenge due to the formation of high‐molecular‐weight ETX micelles and the affinity binding of negatively charged ETX to positively charged molecules such as proteins and—relevant here—IgG. Hence, depending on the end‐product, a great variety of methods—such as adsorption on polycationic ligands, ultrafiltration, and ion‐exchange chromatography—have been developed to achieve this, and depend on the nature of the biopharmaceutical product (Schneier et al., 2020).

Commercial columns to reduce ETX levels are mostly based on affinity chromatography, which selectively adsorbs ETX. Having found that these columns are not efficient enough to reduce ETX contaminations in IgG to permissible levels (providing reductions of only 40% to 60%; data not shown), probably due to the high‐affinity binding of ETX to IgG, we devised an ion‐exchange approach, in which IgG is bound onto the protein A column and ETX is washed off with competing arginine‐containing buffers. Preceding the IgG isolation step, we introduced stringent sanitization measures for devices and solutions used for the IgG isolation, as recommended by regulatory bodies (Grönberg & Hjorth, 2018). Although we successfully established, and describe here, a process to sanitize AK23 IgG consisting of 0.5 M NaOH washing followed by ion exchange with the naturally occurring, nontoxic amino acid arginine, this approach is not suitable for recombinant antibody fragments, such as Px43 (Hammers & Stanley, 2014), that contain a histidine tag and are negatively charged. However, 1% Triton X‐114 can be applied in repeated cycles to quite successfully reduce ETX to 0.2 ‐ 3.5 EU/mg without substantial protein loss, as described by Liu and colleagues (Liu et al., 1997). If ETX stripping is successful, the sanitized antibodies will need to be dialyzed extensively to ensure removal of all detergents.

### Critical Parameters

The first critical parameter in producing low‐ETX IgG comprises the thorough cleaning of all devices and pumps with 0.5 M NaOH, the preparation of all buffers under ETX‐free conditions, and their in‐process testing for ETX contamination after they are run through the column/device and before loading the IgG supernatants. The second critical parameter is the ETX stripping with arginine. In our experience, if the protocols presented here are followed meticulously, the end‐product is compliant with *U.S. Pharmacopeia* standards, containing very low levels of ETX. For example, following this protocol, the 2.2 EU/mg IgG of a AK23 sample was reduced to ≤0.055 EU/mg IgG; this ETX concentration is 1.8 times below the calculated threshold of 0.1 EU/mg IgG, with a net ETX reduction of 40 times, or 97.5%. We recommend purchasing control IgG from a source stating that it was produced under ETX‐free conditions, such as mIgG1 from BioXCell (BE0083, custom‐made ≤0.05 EU/mg).

### Troubleshooting

The in‐process control of ETX in all buffers flowing through the column/device needs to be below in‐process limits (Table 1). If this is not the case, the IgG cannot be charged and the cleaning process using 0.5 M NaOH must be repeated (Basic Protocol 1), with the source of contamination being identified and sanitized. We found that one of the most important sources for ETX contamination can be the pump. It is important to note that the mIgG control also needs ETX testing and must comply with the desired low ETX level. We found it to be even more contaminated than AK23.

### Understanding Results

**Figure 5:**
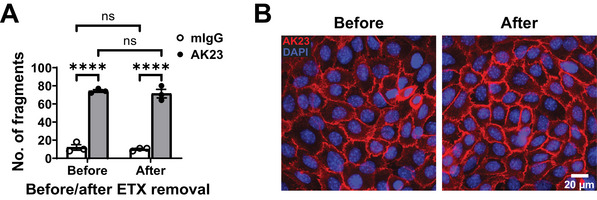
Functional AK23 testing on primary human keratinocytes before and after AK23 IgG sanitization. (**A**) Mechanical disruption of confluent keratinocyte sheet by a KDA. Note that the functional activity of AK23 before and after sanitization is similar. 20 µg/ml AK23 and mIgG1 (BioXCell, BE0083, custom‐made ≤0.05 EU/mg) were used. ns, non‐significant; ****p <.0001; *n* = 3. (**B**) Direct immunofluorescence.

Before performing any experimentation with AK23, it is necessary to confirm the purity and activity of the IgG preparation. First, the purity of AK23 IgG should be confirmed by SDS‐PAGE (Müller et al., 2024) and the absence or low level of ETX with the LAL test (Basic Protocols 1 and 2). Second, the activity of AK23, in terms of inducing loss of intercellular adhesion, is best tested by a keratinocyte dissociation assay (KDA) as compared to mIgG before and after ETX removal (Fig. 5A); we found the optimal concentration for primary human keratinocyte dissociation to be between 1 and 20 µg/ml (see Müller et al., 2024, Fig. 6). We did not detect any change in AK23 activity with the procedure outlined here (Fig. 5).

### Time Considerations

We recommend meticulously following the sanitization procedure for devices and buffers outlined in Basic Protocol 1 and the ETX stripping from IgG described in Basic Protocol 2. In this case, the additional tasks of sanitization and LAL testing will add maximally half a day to 1 day (depending on the flow rate of the column) to the routine IgG isolation protocol (Müller et al., 2024).

### Author Contributions

**Siavash Rahimi**: Conceptualization; data curation; formal analysis; investigation; software; supervision; validation; visualization; writing—original draft; writing—review and editing. **Patrizia Sauta**: Data curation; formal analysis; investigation; methodology; validation; visualization; writing—review and editing. **Monika Edler**: Conceptualization; data curation; formal analysis; investigation; methodology; writing—review and editing. **Elisabeth Locher**: Conceptualization; data curation; formal analysis; investigation; methodology; writing—review and editing. **Marlies Illi**: Conceptualization; data curation; formal analysis; investigation; methodology; writing—review and editing. **Taravat Shojaeian**: Methodology; writing—review and editing. **Luca Borradori**: writing—review and editing. **Thomas Gentinetta**: Conceptualization; data curation; supervision; visualization; writing—review and editing. **William V. J. Hariton**: Conceptualization; data curation; supervision; visualization; writing—review and editing. **Eliane J. Müller**: Conceptualization; data curation; funding acquisition; investigation; methodology; project administration; resources; supervision; validation; visualization; writing—original draft; writing—review and editing.

### Conflict of Interest

The last author is a founder and director of the board of CELLnTEC Advanced Cell Systems AG. We used CELLnTEC media in this study. The following authors are employees of CSL Behring: Thomas Gentinetta, Marlies Illi, Monika Edler, and Elisabeth Locher. All other authors have declared no conflict of interest.

## Data Availability

All data reported are available in this paper. The data used to support the findings of this study, or any additional information required to reanalyze the reported data will be shared upon request.
